# Left atrial veno-arterial extracorporeal membrane oxygenation as a bridge to surgical or percutaneous closure of post-myocardial infarction ventriculoseptal defects: a case series

**DOI:** 10.1093/ehjcr/ytaf095

**Published:** 2025-02-25

**Authors:** Raef Fadel, Jessica Elderkin, Hussayn Alrayes, Gennaro Giustino, Tiberio Frisoli, Mir Babar Basir, Dimitrios Apostolou, Pedro Villablanca

**Affiliations:** Cardiovascular Medicine, Henry Ford Hospital, 2799 W Grand Blvd, Detroit, MI 48202, USA; Wayne State University School of Medicine, 2799 W Grand Blvd, Detroit, MI 48202, USA; Structural Heart Disease, Henry Ford Hospital, 2799 W Grand Blvd, Detroit, MI 48202, USA; Structural Heart Disease, Henry Ford Hospital, 2799 W Grand Blvd, Detroit, MI 48202, USA; Structural Heart Disease, Henry Ford Hospital, 2799 W Grand Blvd, Detroit, MI 48202, USA; Cardiovascular Medicine, Henry Ford Hospital, 2799 W Grand Blvd, Detroit, MI 48202, USA; Cardiac Surgery, Henry Ford Hospital, 2799 W Grand Blvd, Detroit, MI 48202, USA; Structural Heart Disease, Henry Ford Hospital, 2799 W Grand Blvd, Detroit, MI 48202, USA

**Keywords:** Post-myocardial infarct ventricular septal defect, ECMO, Cardiogenic shock, Case report

## Abstract

**Background:**

Post-myocardial infarct (MI) ventricular septal defect (VSD) is a rare and severe complication of an acute MI with high mortality rate. The use of veno-arterial extracorporeal membrane oxygenation (VA-ECMO) as a bridge to surgical or percutaneous repair in cardiogenic shock secondary to post-MI-VSD has been published, but is limited to small case series primarily utilizing surgical ECMO, with the main drawback of potentially increasing afterload and left ventricle pressure, further worsening VSD shunting. Left-atrial VA-ECMO (LAVA-ECMO) can potentially absolve this concern given that it utilizes bi-atrial drainage through a trans-septal fenestrated cannula.

**Case summary:**

Five patients were included in this series, all with VSD secondary to MI, and all managed with LAVA-ECMO as a bridge to repair. Average age was 62 ± 4.2 years, body mass index of 29.4 ± 4.5 kg/m^2^, and left ventricular ejection fraction of 46.6 ± 13.8%. Haemodynamics monitoring pre- and post-LAVA-ECMO demonstrated improvement in right atrial, right ventricular, pulmonary, left atrial, and left ventricular pressures (*Figure 1*). Average time to repair was 7.4 ± 3.9 days. All five patients survived to repair, with four undergoing surgical and one undergoing percutaneous closure. Four out of five patients were decannulated successfully.

**Discussion:**

This case series reports the successful use of LAVA-ECMO as a bridge to MI-VSD repair in patients with cardiogenic shock. Left-atrial VA-ECMO serves as a convenient approach to managing patients with MI-VSD related cardiogenic shock as it is implanted percutaneously, and can be done at the time of shock diagnosis, during right heart catheterization by trained interventionists.

**Learning points**

- Myocardial infarct ventricular septal defect (MI-VSD) is a condition with high mortality if left untreated, especially in the setting of cardiogenic shock.
- Left-atrial veno-arterial extracorporeal membrane oxygenation may serve as a feasible therapeutic option in patients with resultant cardiogenic shock, as a bridge to repair of MI-VSD.

## Introduction

**Figure 1:**
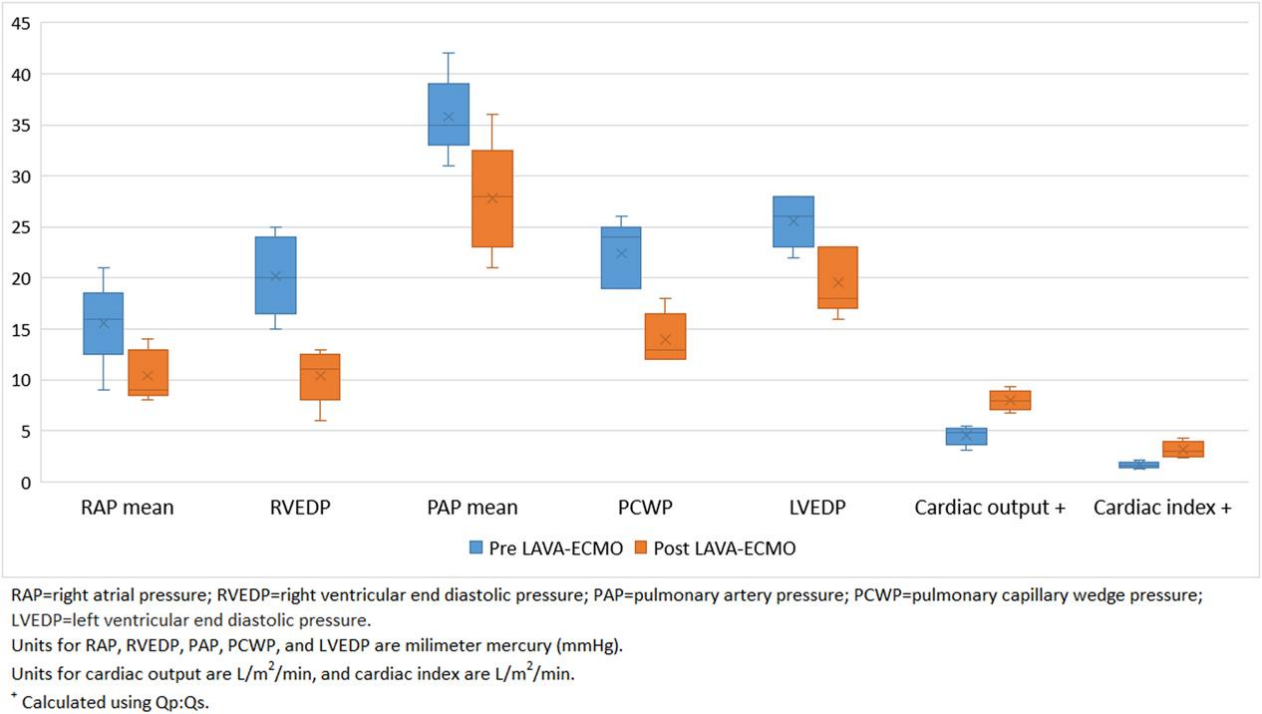
Haemodynamics pre- and post-LAVA-ECMO.

Post-myocardial infarct (MI) ventricular septal defect (VSD) is a rare and severe complication of an acute MI with a mortality rate of 94% without surgical intervention.^1,2^ The utility of veno-arterial extracorporeal membrane oxygenation (VA-ECMO) as a bridge to cardiac surgery or percutaneous valve repair has been successful in haemodynamically stabilizing patients with cardiogenic shock.^2^ The use of VA-ECMO as a bridge to surgical or percutaneous repair in cardiogenic shock secondary to post-MI-VSD has been published, but is limited to small case series primarily utilizing surgical ECMO.^2,3^ Veno-arterial extracorporeal membrane oxygenation can increase afterload, resulting in significant elevations in ventricular filling pressures and increased shunting across the VSD necessitating use of additional venting strategies.^4^ The use of left-atrial VA-ECMO (LAVA-ECMO) utilizes a fenestrated cannula to drain both the left and right atria (*Figure 1*), offering biventricular support without the need for a second large-bore mechanical device, simplifying the procedure, reducing the risk of complications, and minimizing the invasiveness of the treatment.^5^

We present a series of cases with successful preoperative application of LAVA-ECMO in post-MI-VSD as a bridge to definitive intervention. *Table 1* highlights detailed baseline demographics, VSD information, LAVA cannulation specifics, and patient outcomes for each case. The haemodynamic profiles of the patients before and after the utilization of LAVA-ECMO are illustrated in *Table 2* and *Figure 1*.

**Table 1:** Baseline demographics, VSD information, LAVA cannulation, and outcome

	Case 1	Case 2	Case 3	Case 4	Case 5
Characteristics					
Age—years	55	64	62	66	63
Sex	Male	Male	Male	Male	Male
Race	White	White	White	White	White
BMI—kg/m^2^	25.2	29.5	24.9	35.5	32.1
LVEF—%	50	63	45	50	25
Lactate—mmol/L	5.5	1.6	2.5	3.2	1.8
VSD					
Aetiology	MI	MI	MI	MI	MI
Sub-type	Muscular	Muscular	Muscular	Muscular	Muscular
Location^a^	Inferoseptal	Apex	Inferoseptal	Inferoseptal	Apex
Shunt direction^a^	Left-to-right	Left-to-right	Left-to-right	Left-to-right	Left-to-right
Size—cm^a^	1.5 × 1.7	4.3 × 3.2	3.6 × 3.8	1.7 × 1.6	2.2 × 2.7
Qp:Qs^a^	2.1	2.9	3.1	3.6	3.2
MCS details					
Pre-ECMO MCS	None	IABP	mAFP^b^	IABP	IABP
Post-ECMO MCS	LAVA-ECMO	LAVA-ECMO	LAVA-ECMO	LAVA-ECMO	LAVA-ECMO
Inflow cannula					
Location	RFA	LFA	RFA	RFA	RFA
Size—French	17	17	19	19	19
Outflow cannula					
Location	LFV	LFV	RFV	LFV	LFV
Size—French	22	22	23	23	23
Imaging guidance	ICE	ICE	ICE	ICE	ICE
Outcomes					
Days to repair	7	10	11	8	1
Closure technique	Surgical	Surgical	Surgical	Surgical	Percutaneous
Decannulated	Yes	Yes	Yes	Yes	No
In-hospital mortality	No	No	No	Yes	Yes

BMI, body mass index; LVEF, left ventricular ejection fraction; VSD, ventriculoseptal defect; MI, myocardial infarction; ECMO, extracorporeal membrane oxygenation; MCS, mechanical circulatory support; IABP, intra-aortic balloon pump; mAFP, microaxial flow pump; LAVA, left-atrial veno-arterial; RFA, right femoral artery; LFA, left femoral artery; ICE, intracardiac echocardiography; kg, kilogram; m, metre; mmol, millimole; L, litre.

^a^As assessed/measured by TEE.

^b^Impella, Abiomed, Danvers, MA, USA.

**Table 2:** Haemodynamics pre- and post-LAVA-ECMO

	Case 1	Case 2	Case 3	Case 4	Case 5
Haemodynamics					
RAP mean—mmHg					
Pre	16	21	17	16	11
Post	9	12	10	14	8
RVEDP—mmHg					
Pre	20	25	18	23	15
Post	11	12	10	13	6
PAP mean—mmHg					
Pre	36	42	31	35	36
Post	28	28	21	25	29
PCWP—mmHg					
Pre	19	24	20	29	26
Post	12	13	11	15	18
LVEDP—mmHg					
Pre	26	28	33	22	24
Post	23	18	24	15	16
Cardiac output—L/m^2a^					
Pre	4.8	5.5	3.1	4.2	5.1
Post	7.9	9.3	6.8	7.4	8.5
Cardiac index—L/m^2^/min^a^					
Pre	1.4	1.7	1.6	1.3	2.1
Post	2.4	3.0	3.6	2.5	4.3

RAP, right atrial pressure; RVEDP, right ventricular end-diastolic pressure; PAP, pulmonary artery pressure; PCWP, pulmonary capillary wedge pressure; LVEDP, left ventricular end-diastolic pressure; CO, cardiac output; CI, cardiac index; L, litres; m, metres; min, minute.

^a^Calculated using Qp:Qs.

## Summary figure

**Figure:**
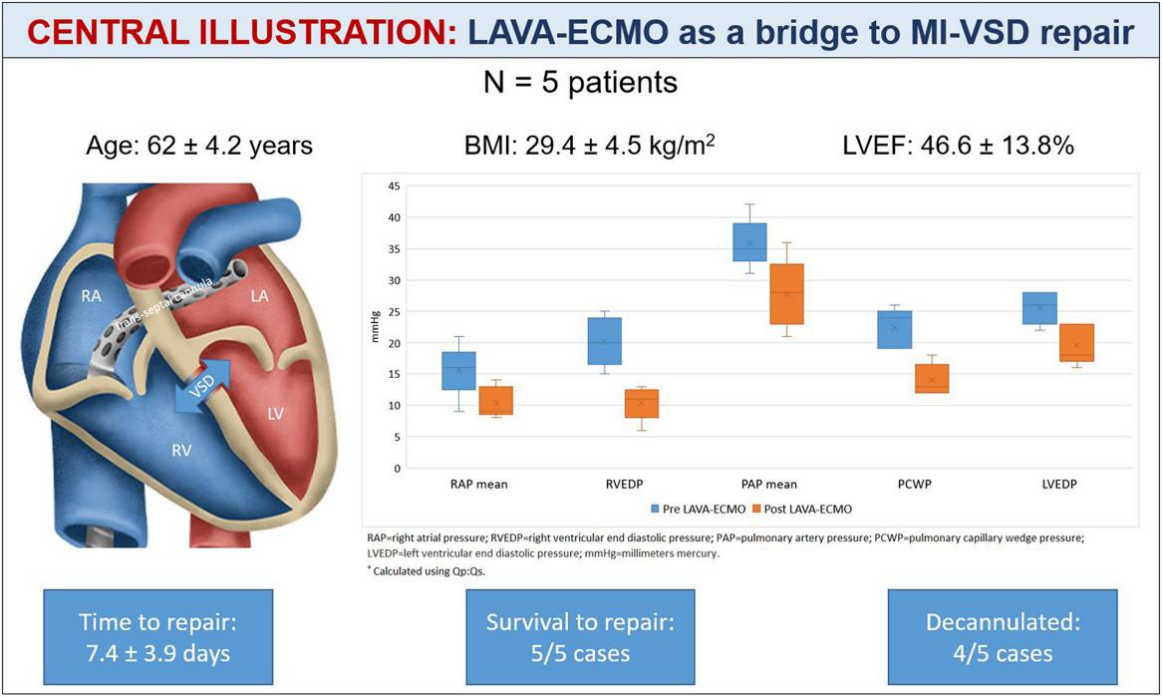


### Patient 1

**Figure 2:**
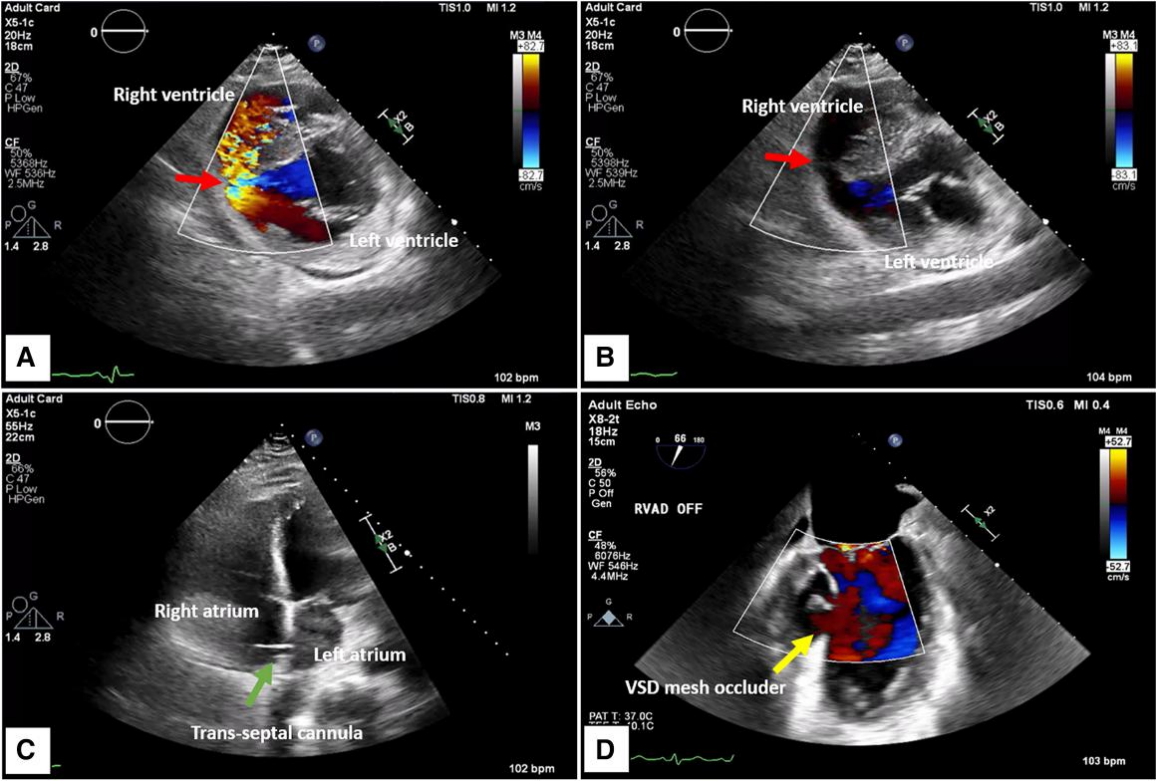
Large mid-inferoseptal VSD in Patient 1 with predominant left-to-right shunting in systole (*A* and *B*, arrow), and the trans-septal LAVA-ECMO cannula well positioned (*C*, arrow). Patient underwent surgical VSD mesh occluder implantation with good post-operative results (*D*, arrow).

A 55-year-old male with no past medical history presented to an emergency department with several hours of chest pain, dyspnoea, and diaphoresis. Initial electrocardiogram (ECG) demonstrated inferior ST-elevation myocardial infarction (STEMI). Coronary angiogram confirmed right coronary artery culprit occlusion. Despite successful stenting, the patient experienced post-operatively hypotension, and transthoracic echocardiogram (TTE) demonstrated a large mid-inferoseptal VSD (*Figure 2A* and *B*). He was transferred to our facility for surgical evaluation and underwent cannulation with LAVA-ECMO for worsening cardiogenic shock (*Figure 2C*). He was taken to the operating room (OR) 7 days later for successful repair of the VSD and closure of iatrogenic atrial septal defect (ASD) (*Figure 2D*). His post-operative course was complicated by hypoxia requiring ECMO support, with decannulation on post-op day (POD) 6. He was discharged to rehab facility on POD 31.

### Patient 2

**Figure 3:**
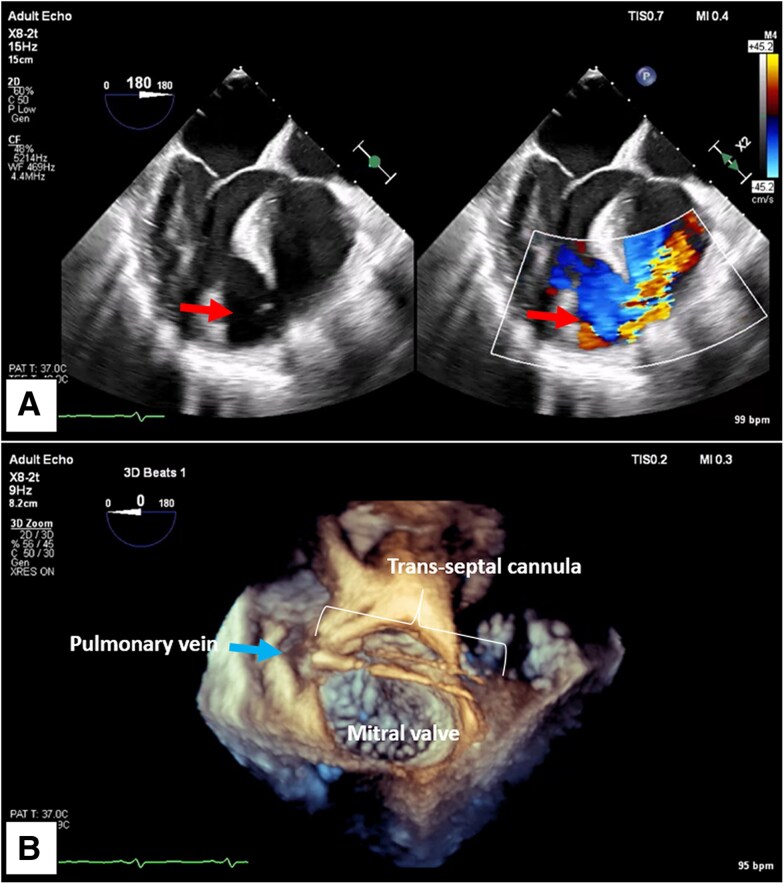
Apical VSD in Patient 2 (*A*, red arrow) with predominant left-to-right shunting. A trans-septal LAVA-ECMO cannula is visualized on 3D from the atrial perspective (*B*, green arrow), crossing into the left atrium and seated in the pulmonary vein (*B*, blue arrow).

A 64-year-old male presented with 5 days of dyspnoea and chest discomfort. Past medical history was significant for coronary artery disease (CAD) and STEMI secondary to left-anterior descending (LAD) coronary artery culprit diagnosed and treated just 18 days prior to presentation. Electrocardiogram demonstrated persistent ST-elevations across the precordial leads, so a TTE was obtained which demonstrated a new VSD with left-to-right shunting (*Figure 3A*). He underwent intra-aortic balloon pump (IABP) insertion and was transferred to our facility for surgical evaluation. A right heart catheterization demonstrated a low cardiac index with elevated pulmonary, right ventricular, and right atrial filling pressures. Intra-aortic balloon pump was exchanged for LAVA-ECMO with improvement in haemodynamics (*Figure 3B*). He underwent successful VSD repair and closure of the iatrogenic ASD 10 days later, however his post-operative course was complicated by multifactorial shock requiring central ECMO cannulation, eventually weaned to veno-venous-ECMO and decannulated on POD 6. He was discharged home on POD 13.

### Patient 3

**Figure 4:**
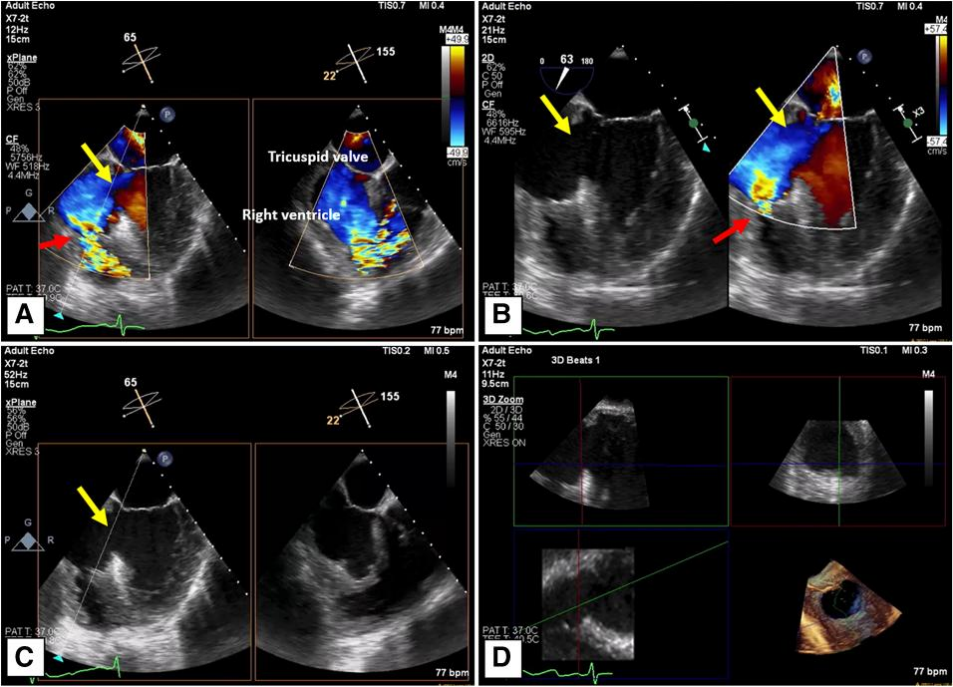
LV septal aneurysm sac with perforation into RV wall seen in mid-systole with colour flow (*A* and *B*), and without colour flow (*C*). 3D reconstruction through the circular orifice measuring 3.55 × 3.79 cm (*D*).

A 62-year-old male with past medical history of diabetes presented with 4 days of exertional chest pain and shortness of breath. Initial ECG demonstrated inferior STEMI, with coronary angiogram demonstrating three-vessel CAD including the left main, and no clear culprit lesion. Transesophageal echocardiogram (TEE) demonstrated left ventricular ejection fraction (LVEF) of 55%, and a large VSD involving an interventricular pseudoaneurysm with perforation into the right ventricle (*Figure 4A–D*). He underwent microaxial flow pump (mAFP) placement and was transferred to our facility for escalation of care. On arrival, he was haemodynamically stable; however, the mAFP was mispositioned in the aortic root. He became hypotensive with lactate elevation to 2.5 mmol/L (ref <2.1 mmol/L). He underwent exchange of the mAFP device for LAVA-ECMO cannulation, with successful VSD repair, iatrogenic ASD closure, and three-vessel coronary artery bypass grafting (CABG) 11 days later given concern for friability of myocardium. An IABP was inserted post-operatively after ECMO decannulation given hypoxia and hypotension, which was subsequently removed on POD 5. He was discharged to a rehab facility on POD 12.

### Patient 4

**Figure 5:**
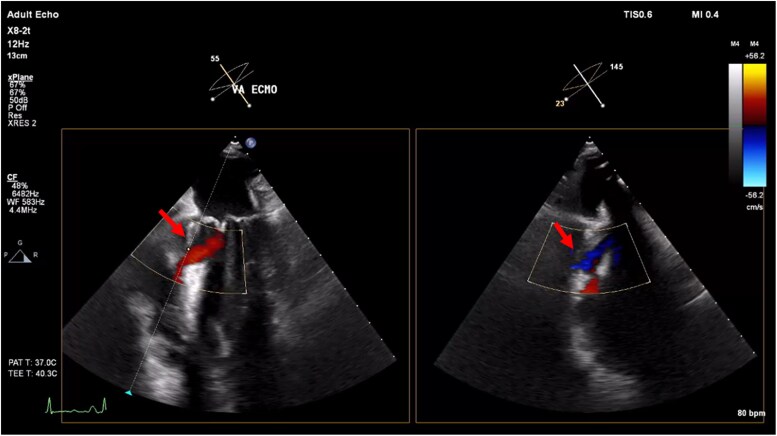
Inferoseptal VSD in Patient 4 depicted by the red arrow, with evidence of bidirectional flow.

A 66-year-old male with no past medical history presented with 3 days of dyspnoea on exertion. On arrival, he was hypotensive and tachycardic. Electrocardiogram demonstrated inferior STEMI, with subsequent coronary angiogram demonstrating three-vessel CAD involving the left main, with no culprit lesion identified. A TEE confirmed presence of a large inferoseptal VSD with bidirectional flow (*Figure 5*). An IABP was inserted for haemodynamic support, however over the next 24 h, he developed worsening haemodynamics which prompted exchange for LAVA-ECMO. He was subsequently taken to the OR 8 days later for successful VSD repair, closure of iatrogenic ASD, and three-vessel CABG. His post-operative course was complicated by haemorrhagic and septic shock, and refractory respiratory failure requiring prolonged intubation. Additionally, he developed acute abdomen secondary to perforated viscus, prompting exploratory laparotomy. He died from cardiopulmonary arrest on POD 44.

### Patient 5

**Figure 6:**
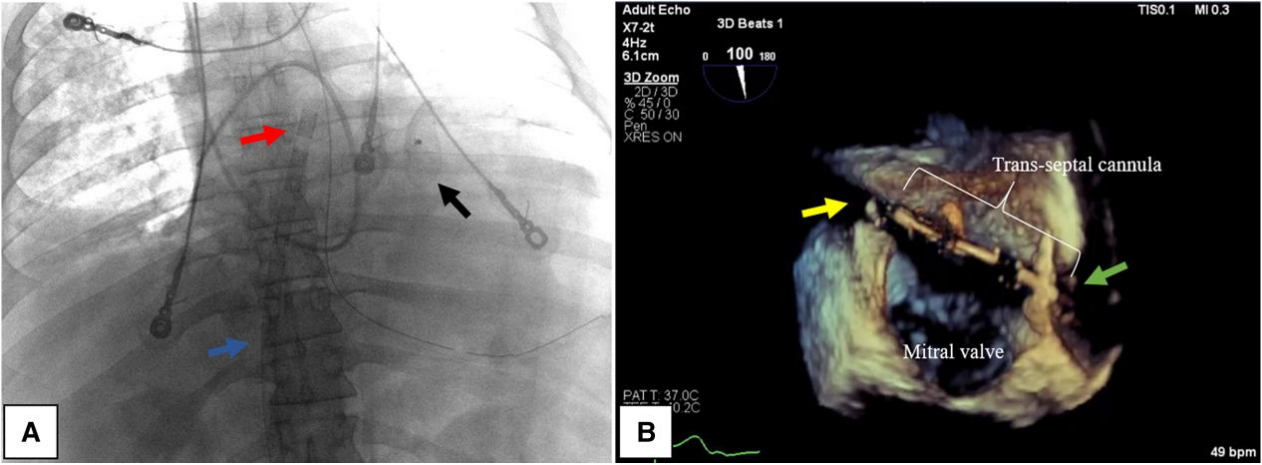
Imaging of the LAVA-ECMO cannulation in Patient 5. On X-ray imaging (*A*) demonstrating the proximal/right atrial (blue arrow) and distal/left atrial (red arrow) segments of the fenestrated cannula. A percutaneously deployed VSD closure device can be seen as well (black arrow). On TEE (*B*), the ECMO cannula can be visualized crossing the interatrial septum (green arrow) into the left atrium, with the distal end seated in the pulmonary vein (yellow arrow).

A 63-year-old male with past medical history of significant tobacco use presented with 3 weeks of dyspnoea on exertion and orthopnoea. On presentation to the emergency department, he was found to be tachycardic and tachypnoeic, and an ECG demonstrated anterolateral STEMI. He underwent coronary angiography which confirmed LAD culprit lesion. A TTE demonstrated presence of apical hypokinesis with thrombus formation, and an apical VSD. An IABP was placed, and he was transferred to our institution for complex intervention and VSD repair. Given low cardiac output and elevated invasive pressures, he underwent LAVA-ECMO cannulation followed by successful percutaneous VSD closure (*Figure 6A* and *B*). His immediate post-operative course was complicated by significant pulmonary haemorrhage which resulted in haemorrhagic shock. His family elected to pursue comfort care on POD 3.

## Discussion

Research investigating haemodynamically stabilizing patients in cardiogenic shock from post-MI-VSD in the acute phase with mechanical circulatory support (MCS) prior to surgical or percutaneous intervention shows that it is effective as a bridge to therapy.^2–4^ Although VA-ECMO is a widely used method of MCS to stabilize patients for surgical repair in the setting of post-MI-VSD, it can increase afterload, thereby exacerbating left ventricular dysfunction, VSD shunt flow, pulmonary capillary pressures, and left ventricular end-diastolic pressure.^4^ Often, VA-ECMO is supplemented with an IABP or an mAFP device to offer biventricular support offloading the left ventricle.^6,7^ However, this approach introduces risk of vascular complications, cerebrovascular accidents, and infection with the introduction of an additional arterial large-bore access.^8^ Left-atrial VA-ECMO aims to vent the left ventricle indirectly, without the need of an accessory large-bore mechanical device.^9^

A VSD introduces significant biventricular strain, especially in the acute phase such as post-MI. Therefore, the goal of stabilization is to offload the heart and augment forward flow. In the setting of circulatory shock, with vasopressors and inotropic agents, this can prove challenging. Mechanical circulatory support plays a critical role in this setting, and LAVA-ECMO is well suited to provide bi-atrial drainage, and indirect biventricular venting, to offload this extra stress. Introduction of an iatrogenic ASD for trans-septal cannulation may seem counterproductive, however these defects are small and are most often left alone without consequence.^9^ The goal of these patients is to bridge to surgical or percutaneous intervention, with decannulation from ECMO. If the ASD requires closure, it can be done at the time of surgery/percutaneous repair. Furthermore, the process of trans-septal puncture and cannulation is safe and feasible.^5,9^ Centres who perform these as part of routine procedures are well equipped and can integrate the practice of LAVA-ECMO cannulation into their MCS programmes with adequate institutional support.

In this case series, cardiogenic shock was defined as a low cardiac output state (accounting for Qp:Qs) with hypotension requiring vasopressors/inotropes, with evidence of end-organ hypoperfusion (kidney function, lactate, etc.).^10^ Despite a normal LVEF, the true cardiac output of these patients given presence of large VSD was low, with resultant elevated filling pressures and need for advanced MCS. The patients in this case series demonstrated elevated filling pressures, with low calculated cardiac output after accounting for shunt fraction, with associated evidence of end-organ hypoperfusion, all of which prompted escalation of treatment.

The use of LAVA-ECMO in a patient with VSD was previously reported successfully.^11^ We build on that publication with a series of five post-MI-VSD cases, in which all patients tolerated the device well and demonstrated improved haemodynamics following the implementation of LAVA-ECMO, facilitating survival to surgical or percutaneous repair. Our case study shows how LAVA-ECMO addresses the dual challenges proposed by post-MI-VSD, namely increased left ventricular work and volume pressures, along with increased preload to the right ventricle.

## Conclusion

While VA-ECMO remains a critical tool in managing post-MI-VSD, the unique benefits LAVA-ECMO offers by more efficiently offloading the heart, minimizing complications, and improving patient outcomes make it a preferred strategy in managing patients with cardiogenic shock due to post-MI-VSD. Continued research focusing on optimizing patient selection criteria, developing protocols for intervention, and monitoring long-term patient outcomes will help to further improve the effectiveness and safety of LAVA-ECMO in post-MI-VSD.

## Data Availability

The data underlying this article are available in the article.

## Lead author biography

Raef Ali Fadel is an ACCFIT at Henry Ford Hospital in Detroit, MI. He has research interests in cardiogenic shock, mechanical circulatory support, and acute myocardial infarction.
